# A meta-analysis of everolimus-eluting stents versus sirolimus-eluting stents and paclitaxel-eluting stents in diabetic patients

**DOI:** 10.1186/s13019-021-01452-8

**Published:** 2021-04-17

**Authors:** Hang Ouyang, Xuehui Zeng, Chunlei Zhang, Linli Song, Jiarui Xu, Zhihui Hou, Siya Xie, Zheng Tao, Jincai He

**Affiliations:** 1 Department of Clinical Laboratory, Shenzhen Traditional Chinese Medicine Hospital, The Fourth Clinical Medical College of Guangzhou University of Chinese Medicine, 1 Fuhua Road, Shenzhen, Guangdong 518033 People’s Republic of China; 2 Department of Vascular Surgery, Affiliated Hospital of Jiangsu University, 438 Jiefang Road, Zhenjiang, Jiangsu 212001 People’s Republic of China

**Keywords:** Sirolimus-eluting stents, Paclitaxel-eluting stents, Everolimus-eluting stent, Diabetes, Meta-analysis

## Abstract

**Objective:**

We performed this meta-analysis to determine which stent among everolimus eluting stents (EES), sirolimus eluting stents (SES) and paclitaxel eluting stents (PES) should be preferred for the treatment of DM patients.

**Methods:**

A systematic search of publications about randomized controlled trials (RCTs) focused on diabetic patients received EES, SES or PES was conducted. We evaluated the following indicators: target vessel revascularization (TVR), target lesion revascularization (TLR), late luminal loss (LLL), stent thrombosis (ST), myocardial infarction (MI), all-cause mortality and cardiac mortality.

**Results:**

EES showed obvious advantages over SES for DM patients, as it induced the lowest rate of target vessel revascularization and target lesion revascularization (TLR) (*p* = 0.04). In addition, EES induced lower in-segment LLL than PSE and SES and lower in-stent LLL than PES in DM patients (all *p* < 0.05). Moreover, EES effectively reduced all-cause mortality compared to SES (RR = 0.71, 95% CI: 0.52–0.99, *p* = 0.04) and MI rates compared to PES (RR = 0.44, 95% CI: 0.26–0.73, *p* = 0.0002). Furthermore, EES could reduce the ST rate compared with both SES (RR = 0.53, 95% CI: 0.28–0.98, *p* = 0.04) and PES (RR = 0.18, 95% CI: 0.07–0.51, *p* = 0.001).

**Conclusion:**

Among those three types of stents, EES should be the first recommended stent for DM patients.

## Introduction

Cardiovascular complications are the main cause of mortality among diabetes mellitus (DM) patients. It has been reported that almost half of DM patients undergo percutaneous coronary intervention (PCI) after diagnosis [1]. Moreover, although little difference between patients with and without DM was observed in the early stage after PCI, patients with DM often had a worse prognosis and higher rates of restenosis, multivessel revascularization and revascularization than those without DM [2–4].

Some randomized controlled trials (RCTs) found that EES and SES showed comparable overall safety and efficacy, and both were better than PES [5, 6]. For DM patients, as reported by Conder in 2017, EES has significant advantages over other stents, including SES and PES, and therefore is recommended as the priority choice of DM patients undergoing PCI. However, another RCT demonstrated that EES had an increased trend in the rate of target lesion revascularization (TLR) than PES for DM patients who received insulin treatment [7]. Therefore, we performed this meta-analysis in order to evaluate whether EES is indeed a better stent for DM patients than SES and PES,

## Materials and methods

### Search strategy

In November 2019, we searched the PubMed, Cochrane, and EMBASE databases and CNKI, Wanfang, and Clinicaltrials.gov for all randomized controlled trials (RCTs) comparing EES with SES or PES. Complex search strategies were formulated and conducted after we selected the following Mesh terms as keywords: drug-eluting stents, everolimus, sirolimus, paclitaxel, first-generation, diabetic, and diabetes. An extensive search of the ISI Web of Science database using cross-references from the eligible articles and relevant reviews was also conducted. The language of the articles was restricted to English and Chinese.

### Selection criteria

RCTs about EES vs SES or EES vs PES that met the following inclusion criteria were included in the present study: (1) patients were diagnosed with diabetes; (2) clinical outcomes were reported; and (3) follow-up data lasted more than half a year. RCTs were excluded if they met any of the following criteria: (1) retrospective or nonrandomized trials; (2) some patients were not diabetic; (3) SES vs PES or indirect comparison between EES, SES or PES.

### Study enrolment and data extraction

The two researchers (H.O. and X.Z.) independently performed the literature search and extraction of patient data, including baseline data and postoperative imaging data, and other follow-up results using predetermined standardized tables. Target vessel revascularization (TVR), target lesion revascularization (TLR), late luminal loss (LLL), stent thrombosis (ST), myocardial infarction (MI), all-cause mortality and cardiac mortality were recorded. If there was a disagreement between the two researchers, an independent third person resolved the problem according to the Cochrane collaboration [8]. If there were any incomplete or suspicious research data, we tried to resolve the issue by contacting the authors. We used the Cochrane Risk Bias Evaluation Tool to assess the quality of the included articles .

### Statistical analysis

We used RevMan v5.3 (Copenhagen, The Nordic Cochrane Centre) to analyse all the collected results. Continuous results were recorded as dichotomous data, while LLL was recorded as the standardized mean difference (SMD). To avoid the influence of heterogeneity of the included trials on overall effects, we computed the risk ratios (RRs) and 95% confidence intervals (CIs) with two-sided *P*-values for all results. Statistical significance was defined as *P* < 0.05. The heterogeneity of the included RCTs was assessed using Higgins and Thompson’s I^2^ statistic. When I^2^ > 50%, the heterogeneity of the RCT was considered high. All analyses were conducted under PRISMA guidelines [9]. Since we trying to confirm whether the application of EES in diabetic patients has obvious advantages, we also combined SES and PES as the first-generation stent for research to ensure that the conclusion is foolproof.

### Registration of the study protocol

The protocol for this study was prepared prior to the start of the study and was registered in PROSPERO with identification number CRD42019130007.

## Results

### Selected studies and characteristics

**Fig. 1:**
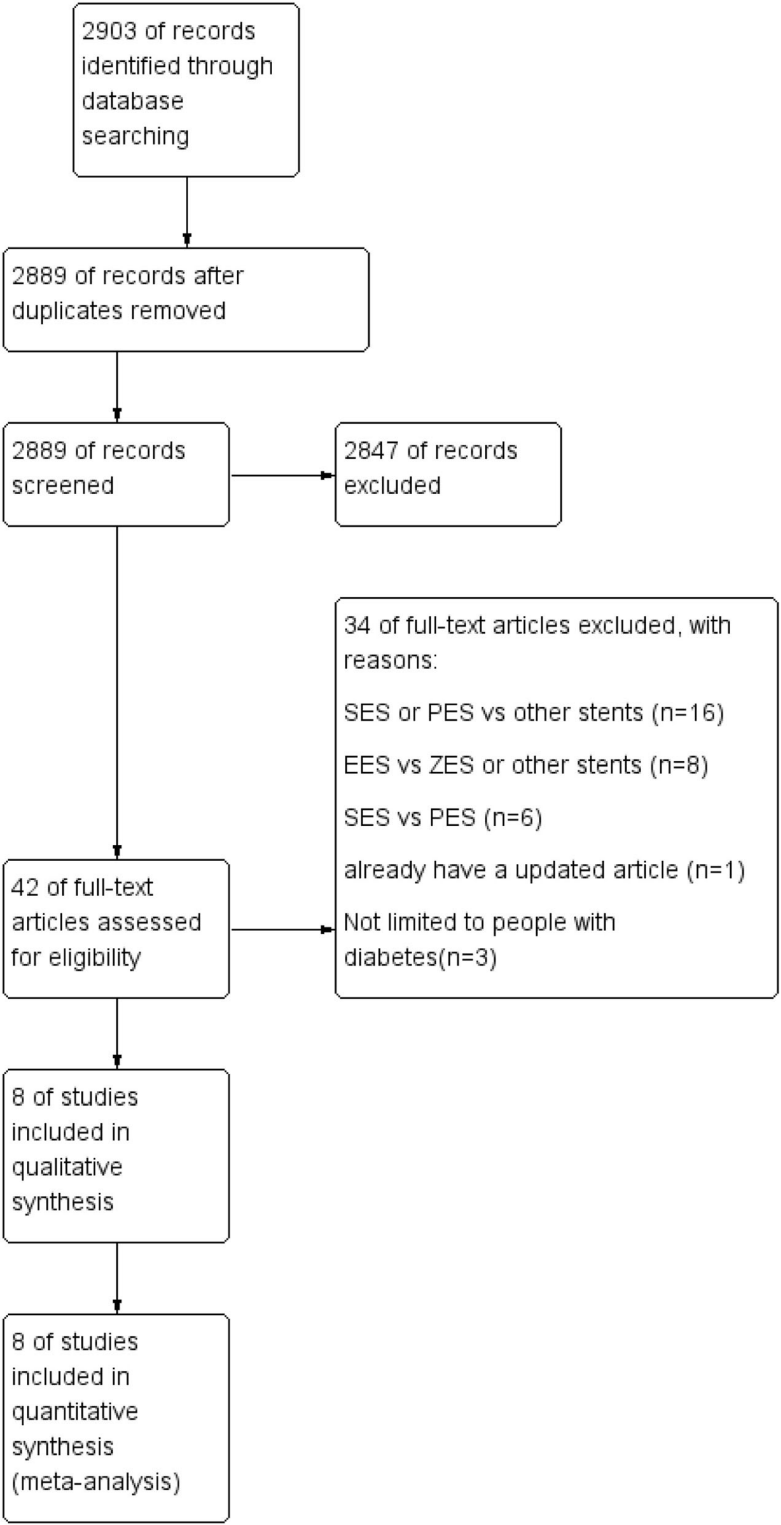
Flowchart of the selection strategy and inclusion/exclusion criteria in the current meta-analysis. EES: everolimus-eluting stents, ZES: zotarolimus-eluting stents, PES: paclitaxel-eluting stents, SES: sirolimus-eluting stents

**Fig. 2:**
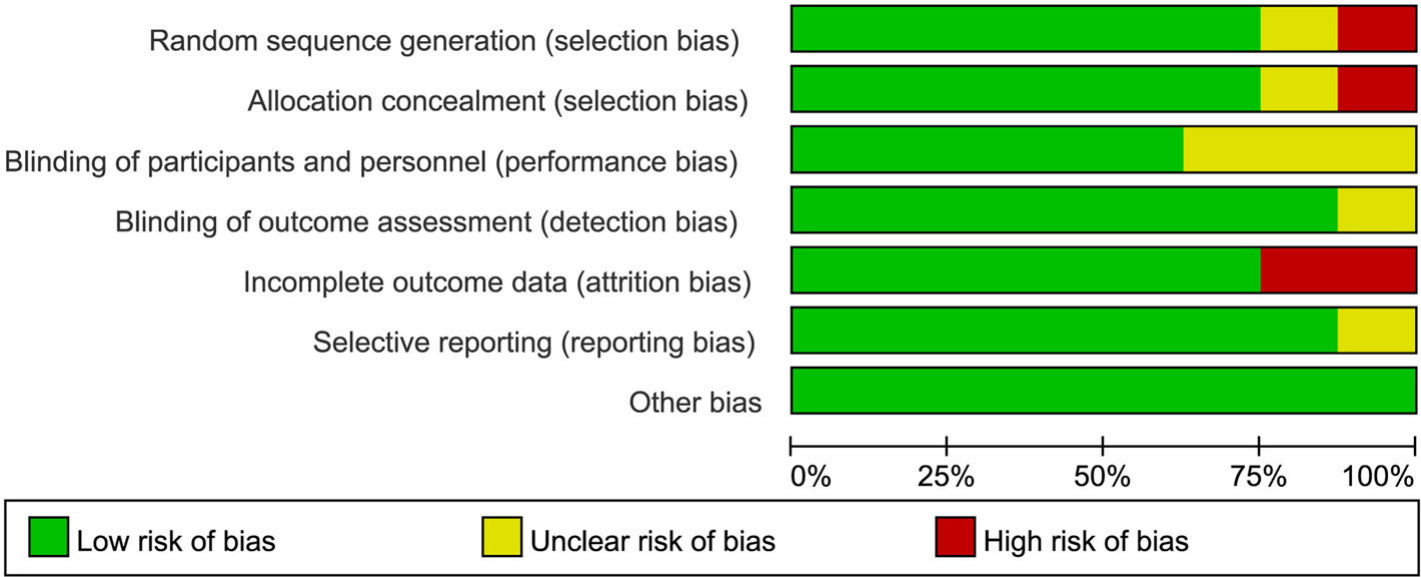
Risk of bias graph

**Fig. 3:**
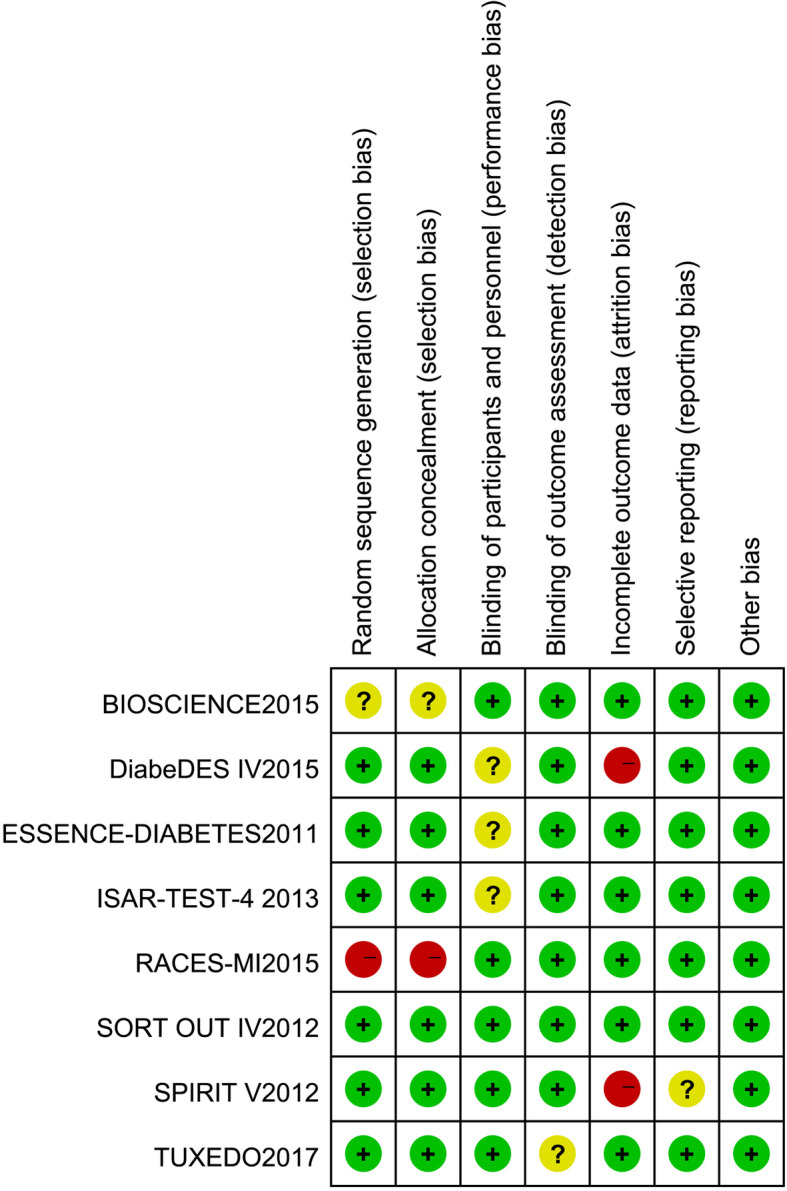
Risk of bias summary

The primary search identified 2903 articles, and 8 [7, 10–16] met the inclusion criteria and were therefore included in our study (Fig. 1). A total of 4047 DM patients were included, 1898 of whom were randomly located in the EES vs SES group, and the others were located in the EES vs PES group (Table 1). All basic features of the patients in each group are shown in Table 1. All patients were treated with DAPT for 6 months or 12 months under the protocol of treatment or research guidelines. The average age of the patients ranged from 58 to 68 years and the proportion of men ranged from 43 to 76% in the included RCTs. In addition, the incidence of acute coronary syndrome ranged from 31.9 to 53%. Furthermore, three of the included clinical trials reported 12-month follow-up data [7, 10, 12], one of them had 18-month follow-up data [11], and the others had follow-up data for ≥24 months [13–16]. The duration of DAPT (dual antiplatelet therapy, DAPT) treatment was 6 months in two trials [7, 13] and 12 months in the others. The results of bias assessment are shown in Figs. 2 and 3.

**Table 1:** Baseline characteristics of the included trials

Trial	Published years	Comparison arms	Sample size	Follow-up, months	DAPT duration, months	Mean age, years	Male, %	Insulin use, %	ACS, %	Primary endpoint	Current smoker, %	BMI
BIOSCIENCE	2015	EES vs SES	229/257	12	12	68	76	32.9	45	Target lesion failure, Cardiac death, TV-MI, TLR	22	29.5
DiabeDES IV	2015	EES vs SES	108/105	48	12	63	NR	NR	31.9	In-stent late luminal loss	23	29.5
RACES-MI	2015	EES vs SES	64/68	36	12	61	68	37.1	NR	MACE	26	NR
ESSENCE-DIABETES	2011	EES vs SES	149/151	12	12	64	59	15.3	42	In-stent late loss	24	NR
ISAR-TEST-4 Trial	2013	EES vs SES	184/193	36	6	68	74	32.4	40	Cardiac mortality, TV-MI, TLR	14.3	NR
SORT OUT IV	2012	EES vs SES	194/196	18	12	64	74	32.1	33	Cardiac mortality, MI, ST, TVR	22.8	NR
SPIRIT V	2012	EES vs PES	215/104	12	6	65	43	17.2	37	In-stent late loss	16.4	NR
Tuxedo	2017	EES vs PES	916/914	24	12	58	75	40.8	53	TVF, TV-MI, TVR	15	26

*DAPT* dual antiplatelet therapy, *ACS* acute coronary syndrome, *BMI* body mass index, *EES* everolimus-eluting stents, *PES* paclitaxel-eluting stents, *MI* myocardial infarction, *TLR* target-lesion revascularization, *MACE* major adverse cardiac events, *TV* target-vessel, *TVR* target-vessel revascularization, *ST* stent thrombosis, *TVF* target vessel failure *NR* not reported

### TVR and TLR

**Fig. 4:**
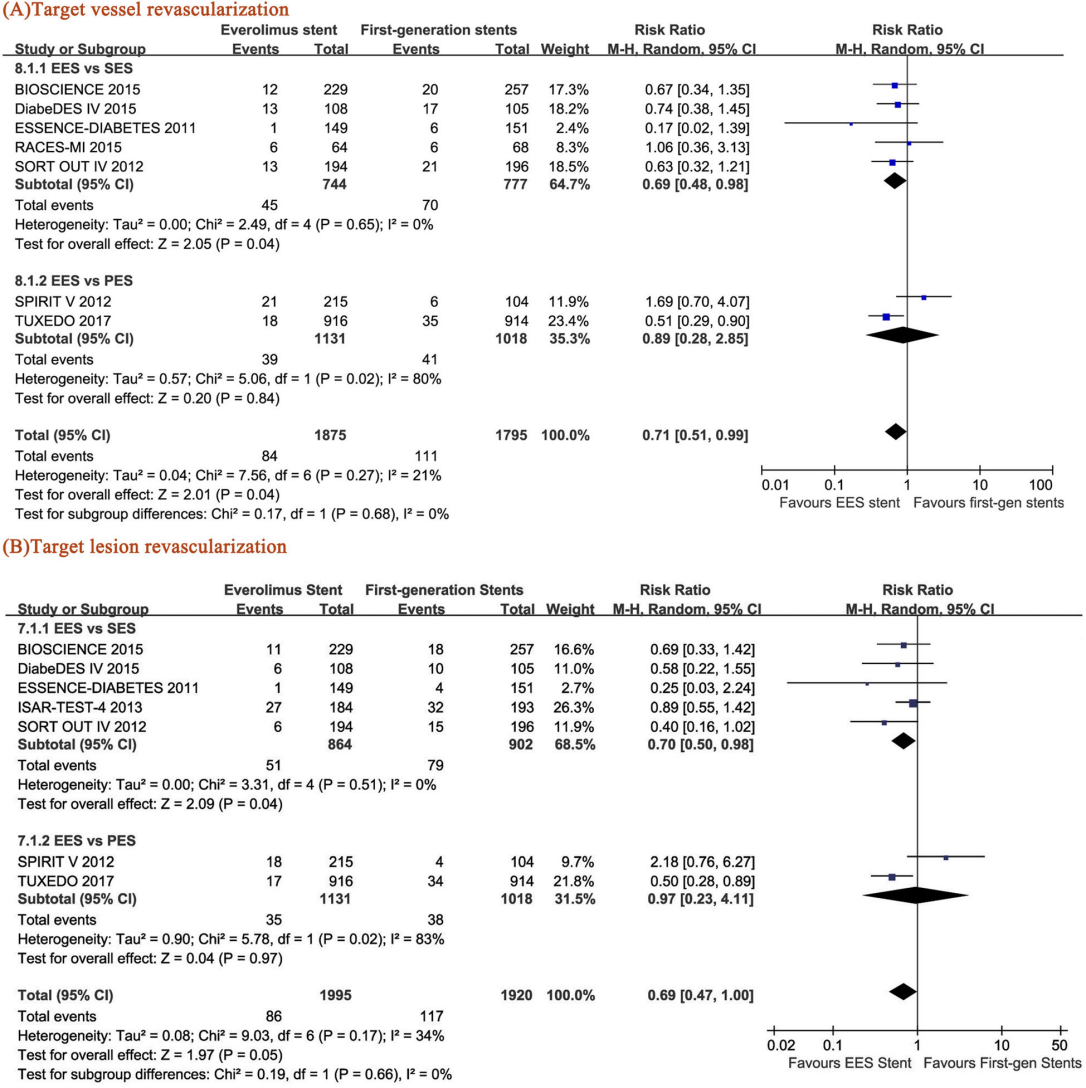
Forest plots of the pooled risk ratios for (**a**) target vessel revascularization (TVR) and (**b**) target lesion revascularization (TLR)

TVR and TLR were employed in this analysis as indicators for the effectiveness of PCI. The TVR of the patients with EES was significantly lower than that of the patients with SES (RR = 0.69, 95% CI: 0.48–0.98, *p* = 0.04) and that of the pooled data of SES and PES (RR = 0.71, p = 0.04) (–. 4A). In addition, EES induced a lower TLR rate than SES (RR = 0.70, 95% CI: 0.50–0.98, p = 0.04). However, although a reduced trend of TLR was observed in the DM patients with EES compared with the pooled data of the SES-treated and PES-treated patients (RR = 0.69, 95% CI: 0.47–1.00, *p* = 0.05), no statistical significance was obtained between the patients with EES and PES (RR = 0.97, 95% CI: 0.23–4.11, *p* = 0.97) (Fig. 4b).

### In-segment LLL and in-stent LLL

**Fig. 5:**
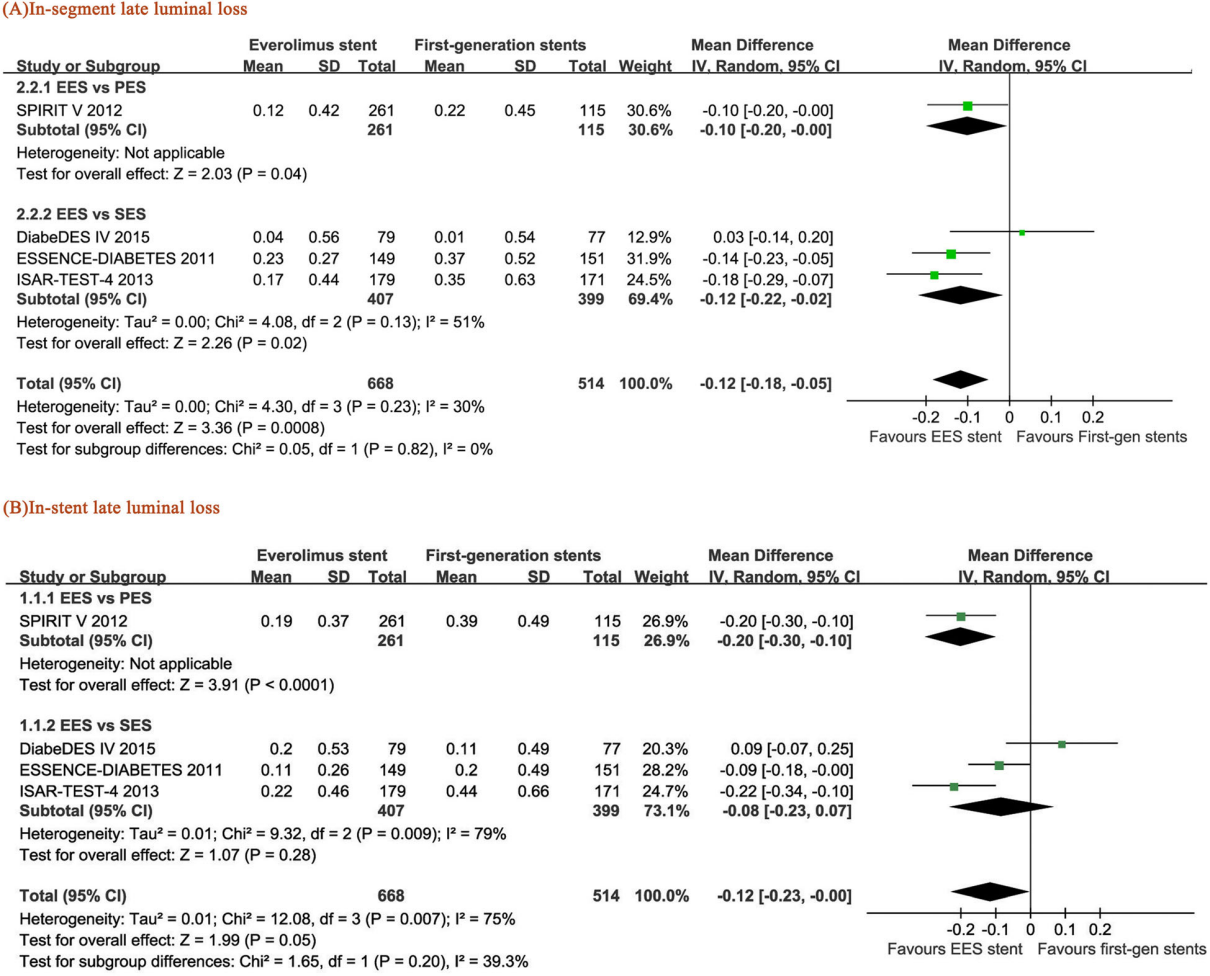
Forest plots of the pooled risk ratios for (**a**) in-segment late luminal loss (in-stent LLL) and (**b**) in-stent late luminal loss (in-stent LLL)

Four of the eight RCTs reported the length of late luminal loss. EES induced lower in-segment LLL than SES (RR = − 0.12, 95% CI: − 0.22– − 0.02, *p* = 0.02) and PES (RR = -0.10, 95% CI: − 0.20– − 0.00, *p* = 0.04) and the pooled SES and PES (RR = − 0.12, 95% CI: − 0.18– − 0.05, *p* = 0.0008) (Fig. 5a). A high level of statistical heterogeneity was found in the analysis (I^2^ = 79% for the EES vs SES group). Similarly, less in-stent luminal loss was observed in the EES-treated patients than in the PES-treated patients (RR = − 0.2, 95% CI: − 0.3– -0.1, *P* < 0.0001), and there was also a reduced trend in the pooled data of SES and PES (RR = − 0.12, 95% CI: − 0.23– − 0.00, *p* = 0.05) (Fig. 5b).

### All-cause mortality and cardiac mortality

**Fig. 6:**
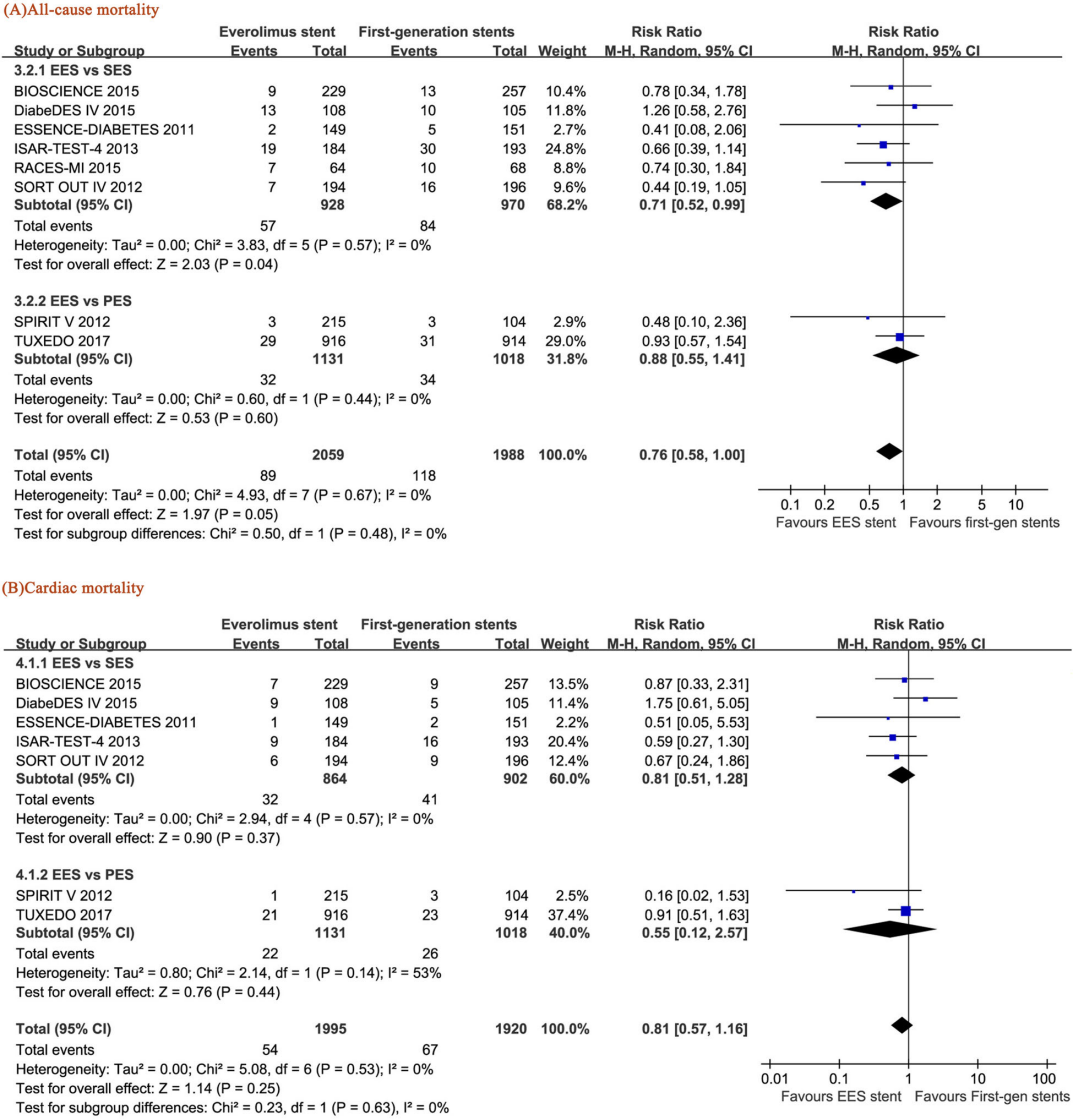
Forest plots of the pooled risk ratios for (**a**) all-cause mortality and (**b**) cardiac mortality

EES significantly reduced all-cause mortality compared with SES (RR = 0.71, 95% CI: 0.52–0.99, *p* = 0.04) but not PES (RR = 0.88, 95% CI: 0.55–1.41, *p* = 0.60) (Fig. 6a) and showed a decreasing trend compared with the pooled data of overall first-generation DES including SES and PES (RR = 0.76, 95% CI: 0.58–1.00, p = 0.05) (Fig. 6). In addition, no difference in cardiac mortality was found between EES and SES (RR = 0.81, 95% CI: 0.51–1.28, *p* = 0.37) or EES and PES (RR = 0.55, 95% CI: 0.12–2.57, *p* = 0.44) (Fig. 6b). A low level of statistical heterogeneity was found in the analysis (I^2^ = 0% for both all-cause mortality and cardiac mortality comparisons).

### Myocardial infarction and stent thrombosis

**Fig. 7:**
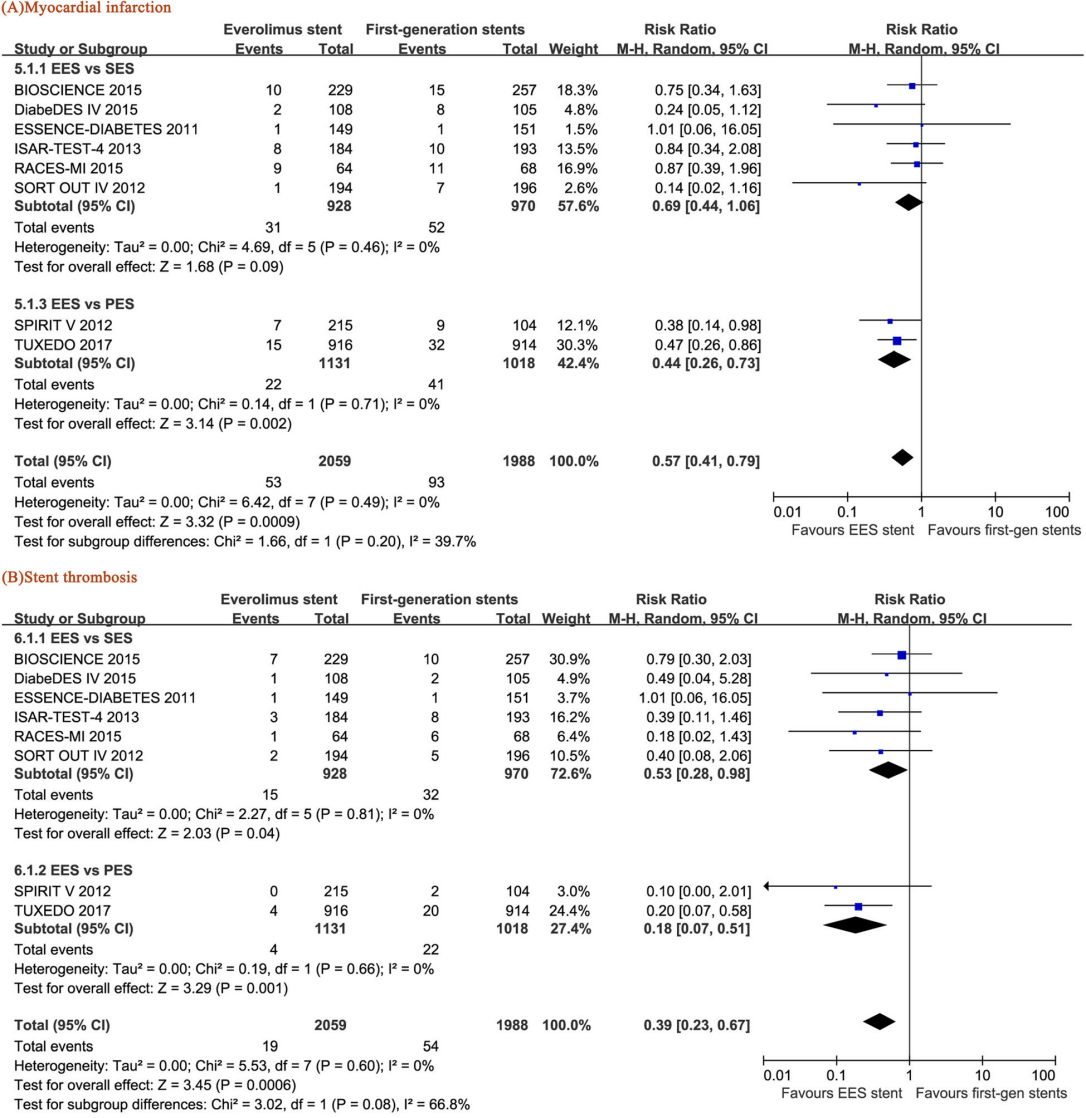
Forest plots of the pooled risk ratios for (**a**) myocardial infarction and (**b**) stent thrombosis

A lower MI rate was observed in the EES-treated patients than in the PES-treated patients (RR = 0.59, 95% CI: 0.35–0.98, *p* = 0.04). Furthermore, the MI rate of the EES-treated patients was lower than that of the pooled SES and PES data (RR = 0.65, 95% CI: 0.49–0.87, *p* = 0.003) (Fig. 7a). In addition, EES showed promising efficacy in the prevention of ST, as it induced a lower ST rate than SES (RR = 0.53, 95% CI: 0.28–0.98, p = 0.04) or PES (RR = 0.18, 95% CI: 0.07–0.51, *p* = 0.001) or the pooled SES and PES (RR = 0.39, 95% CI: 0.23–0.67, *p* = 0.0006) (Fig. 7b).

### Sensitivity and subgroup analysis

After we used the fixed effect model, some of our previously nonsignificant results became statistically significant, such as EES vs SES for in-stent LLL and myocardial infarction and EES vs PES for ST. Therefore, we performed subgroup analysis for TLR and ST according to the DAPT duration (< 12 months or = 12 months), the follow-up time (< 24 months or ≥ 24 months) and insulin application. For the comparison of the EES-treated versus PES-treated patients and the EES-treated versus SES-treated DM patients, the TLR rate was lower in the subgroup with a 12-month DAPT duration than that with a DAPT duration less than 12 months (*p* = 0.01). Nevertheless, no significant difference in the TLR rate and the ST rate was found between the remaining subgroups (all *p* > 0.05) (Table 2).

**Table 2:** Subgroup analyses based on the data of TLR and stent thrombosis; CI - confidence interval, DAPT - dual antiplatelet therapy, RR - risk ratio, TLR - target lesion revascularization

	Stent thrombosis			TLR		
Subgroups	No. of studies	RR (95% CI)	Interaction *P* value	No. of studies	RR (95% CI)	Interaction P value
≤30% patients with insulin therapy	2	0.34 (0.03, 3.43)	0.91	2	0.92 (0.12, 7.32)	0.74
> 30% patients with insulin therapy	5	0.39 (0.22, 0.70)		4	0.65 (0.46, 0.91)	
DAPT duration = 6 months	2	0.32 (0.09, 1.05)	0.69	2	1.09 (0.67, 1.76)	0.01
DAPT duration = 12 months	6	0.42 (0.23, 0.75)		5	0.50 (0.34, 0.74)	
< 24-months follow-up	4	0.61 (0.28, 1.30)	0.12	4	0.72 (0.32, 1.58)	0.9
≥24-months follow-up	4	0.26 (0.13, 0.55)		3	0.68 (0.46, 1.00)	

### Heterogeneity analysis

High heterogeneity was found in the EES vs PES group when we performed the analysis of TLR and TVR (Fig. 4). Since only two studies met the included criteria of this group, sensitivity analysis was not applicable. In addition, relatively high heterogeneity was found in the EES vs SES group when evaluating in-stent LLL. However, no change in the merged effect of in-stent LLL and in-segment LLL (both *P* < 0.05) was observed in the sensitivity analysis after removing RCT DiabeDES IV [14]. Furthermore, we could not find the source of heterogeneity after carefully reviewing the RCT DiabeDES IV [14].

## Discussion

To date, drug-eluting stents have been recommended as the primary choice for patients with coronary heart syndrome undergoing PCI because they have a better performance than bare metal stents (BMS) in reducing the recurrence rate, myocardial infarction rate, and inflammatory response in patients, which would therefore prolong patient survival.

In the past, sirolimus and paclitaxel were among the most common drugs for DES. Everolimus, an analogue of sirolimus, inhibits FRAP protein expression and intima proliferation. To date, many studies have demonstrated that EES shows advantages over SES and PES in long-term prognosis and has become the most widely used stent in Europe and the United States [17, 18].

DM is an independent predictor of early ST that could induce some unique cardiovascular changes in patients, including intimal dysfunction, endothelial hyperproliferation, and platelet dysfunction, thus impairing vascular vasodilation and finally leading to poorer clinical outcomes [19]. Thus, the choice of stents is further complicated in DM patients. Many previous studies have found that compared to PES, SES induced lower levels of LLL and TLR and less endometrial hyperplasia in diabetic patients [20–22], indicating that the efficacy and safety of different kinds of DES for DM patients could vary.

More recently, according to large-scale meta-analysis and reviews, EES showed better efficacy and safety than other DES for patients due to their better postoperative blood flow reconstruction and lower occurrence of TLR and ST [23, 24]. However, the antiproliferative effect of EES could be attenuated due to the high glucose status in diabetic patients [18].. As more complications and higher mortality were found in DM patients undergoing PCI than other patients undergoing PCI, identification of a more suitable stent is urgently needed.

Thus, our current study focused on which kind of DES among EES, PES and SES should be first recommended for DM patients. This meta-analysis revealed that EES is more effective and safer than SES and PES in the treatment of diabetic patients. Compared with SES, EES reduced the occurrence of TLR and TVR by 30 and 31%, respectively. When the DAPT duration was 12 months, the reduction in TLR was more obvious (34%). LLL has been considered another indicator of the anti-restenosis effect and effectiveness of stents after PCI [25–27]. In-stent LLL and in-segment LLL reflect the extent of intimal hyperplasia and the antiproliferative capacity of stents; thus, both of them could be used as predictors of restenosis [28, 29]. According to our results, EES induced 20% less in-stent LLL than PES and 12 and 10% less in-segment LLL than SES and PES, respectively. These data suggested a better anti-restenosis effect of EES. In addition, EES has shown outstanding long-term advantages over SES in the treatment of DM patients, as it significantly reduced all-cause mortality by 29%. Moreover, EES reduced the rate of MI by 56% compared with PES. Furthermore, the ST rate was 47 and 72% lower in the EES-treated patients than in the SES-treated and PES-treated patients, respectively.

Of note, MACE has been used as the main indicator of safety in some previous meta-analyses that focused on the differences between first- and second-generation DES [30]. However, as there is no clear and unified standard for MACE, the results of RCTs could be substantially different if different judgement standards of MACE were employed [31]. Therefore, we did not employ MACE as a safety indicator in this meta-analysis.

### Limitations

According to the Cochrane Handbook for Systematic Reviews of Interventions, it is recommended that at least 10 studies be included in the funnel plot; otherwise, it would be not sufficient to objectively evaluate the symmetry of the funnel plot. Thus, as the present study only included 8 RCTs, the funnel plot was not used.

Furthermore, we could not find the source of heterogeneity after carefully reviewing the RCT DiabeDES IV [14]. We presumed that this hetrogeneity may be due to the limited number of studies included. Therefore, we used random effect models to ensure that the research conclusions can be interpreted with caution. Subgroup analysis based on DAPT duration demonstrated that TLR was significantly higher in the subgroups with a DAPT duration of 12 months than in those with DAPT duration of 6 months (*p* = 0.01) (Table 2). However, due to the limited number of studies and samples included, large-scale studies are needed to further clarify the preliminary conclusions in this report. Moreover, we did not have data about insulin treatment for the patients. However, studies have found that although a lower overall TLR rate has been demonstrated in the EES group than in the PES group during the 2-year follow-up, only the DM patients without insulin treatment could take advantage of EES [10, 18].

As these above limitations might lead to some research biases, more RCTs with abundant sample numbers are urgently needed for a more convincing research conclusion.

## Conclusion

Compared with nondiabetic patients, DM patients have a higher risk of severe multivascular coronary blood flow disorders and worse prognosis. The present meta-analysis proved that EES has better safety and efficacy for DM patients than SES and PES and showed good potential to be the first choice of DES for those patients.

## Data Availability

Not applicable.

## Abbreviations

EES: Everolimus-eluting stent
SES: Sirolimus-eluting stents
PES: Paclitaxel-eluting stents
CVD: Coronary vascular disease
LLL: Late luminal loss
MI: Myocardial infarction
ST: Stent thrombosis
